# Patient prioritisation for hospital pharmacy services: current approaches in the UK

**DOI:** 10.1136/ejhpharm-2020-002365

**Published:** 2020-12-01

**Authors:** Aseel S Abuzour, Gillian Hoad-Reddick, Memona Shahid, Douglas T Steinke, Mary P Tully, Steven David Williams, Penny J Lewis

**Affiliations:** 1 Division of Pharmacy and Optometry, The University of Manchester, Manchester, UK; 2 Retired, Patient and Public Involvement member, Manchester, UK; 3 Manchester University NHS Foundation Trust, Manchester, UK; 4 Pharmacy, Westbourne Medical Centre, Bournemouth, UK

**Keywords:** clinical pharmacy, health services administration and management, pharmacy management (organisation and financial), pharmacy management (personnel), risk management

## Abstract

**Objectives:**

To survey and explore current approaches to deployment of pharmaceutical care prioritisation tools in acute hospitals in the UK.

**Methods:**

A national online survey was circulated electronically to chief pharmacists of hospitals to determine if they use a prioritisation tool or process. Where such mechanisms exist, respondents were invited to participate in a semistructured telephone interview to explore the development, evaluation and application of their tool and share relevant documentation. Interviews were transcribed and thematically analysed.

**Results:**

Seventy hospitals (70/130) used a tool or process to prioritise clinical pharmacy services. Thirty-six interviews were conducted, and two were excluded. The majority of tools had been developed in-house. Few hospitals had undertaken formal evaluations of their prioritisation tool. Pharmacy prioritisation tools ranged in complexity and often included a combination of pharmacy service prioritisation, such as medicines reconciliation, and a section to assign an individual patient prioritisation level. Determining the priority of a patient based on the identification of set indicators instilled confidence in pharmacists by ensuring they were not missing high-risk patients. Electronic prioritisation tools were especially useful at retrieving real-time data to prioritise workload, improving workflow and ensuring continuity in patient care. Drawbacks of using prioritisation tools included lack of tool sensitivity across certain specialties and time spent using the tool if not all information was accessible.

**Conclusions:**

Prioritisation tools were seen to be useful for prioritising workload and ensuring the right patients are seen at the right time. As few hospitals had formally evaluated their tools, it is important to rigorously and systematically develop an evidence-based prioritisation tool that is both useable and acceptable. Further research to evaluate such tools would be needed to ensure it improves patient health outcomes and efficiency in pharmacy services.

## Introduction

The ever-increasing pressure on healthcare services in the UK caused by multiple complex issues has driven new and more efficient ways of working. Better use of the healthcare workforce has a vital role in increasing efficiency in the UK National Health System (NHS).^1^ A report on healthcare productivity in England stated that hospitals should ‘grasp the use of their resources more effectively, the most important of which is their people’.^2^ One approach to increasing efficiency has been the prioritisation of patients for healthcare services. The National Quality Board publication^3^ outlines the importance of supporting NHS staff to ‘deliver the right staff, with the right skills, in the right place at the right time’. Some healthcare professions, such as nursing, have invested in evidence-based decision support tools to classify the severity of a patient’s condition, as one method to prioritise care delivery to patients^4^ and inform workforce decisions.^5^

Pharmacists play a key role in medicines optimisation, addressing drug-related problems and preventing adverse drug events.^6 7^ However, like other NHS healthcare professions, hospital pharmacists are under pressure to meet the needs of increasing numbers of patients without increasing numbers of staff, alongside ambitions to deliver the same level of service 7 days of the week.^8^ Current standards for hospital pharmacy in Great Britain highlight the need for systems to identify patients most likely to benefit from clinical pharmacy support,^9^ yet there is currently no national standard or process to prioritise patients for such input. The pharmacy team is expected to reconcile all patients’ medicines within 24 hours of hospital admission,^9^ but achieves this for only 68% of inpatients.^10^ This could pose a risk to those patients requiring urgent pharmaceutical input, such as interventions for time-critical medicines or correction of important prescribing errors.

Intelligence about which patients require the most detailed or prompt pharmacy input would be valuable when delivering safe and efficient pharmacy services. Prioritisation is usually based on identification of specific factors that make a patient more likely to experience adverse outcomes. In pharmacy, such factors include high-risk medicines, major organ failure and complex medication regimens.^11^ Tools to detect the presence of these factors could determine which patients would benefit from timely or detailed/specialist pharmacist input.

A systematic review of patient prioritisation for pharmaceutical care in hospital found most risk assessment tools were developed in Europe, with nine from the UK.^12^ The review concluded that, despite the tools being heterogeneous, they were all perceived by their users as beneficial in identifying patients at most risk of drug-related problems, and therefore helpful to guide the provision of resource-limited pharmacy services. Despite the small number of published UK studies, anecdotally it is known that many more hospitals employ methods to direct pharmacy services to those patients in most need.^13^ The aim of this study was to survey and explore current approaches to the deployment of pharmaceutical care prioritisation tools or processes in acute NHS hospitals in the UK.

## Methods

A brief survey was followed by semistructured telephone interviews with a hospital pharmacy team representative.

### Survey

An invitation to complete a brief online survey was sent electronically to chief pharmacists of UK NHS health boards and trusts (see online supplemental material for definition of terms in this manuscript specific to healthcare in the UK) to determine if they used a prioritisation tool or process, if the tool was developed by their trust or adapted/adopted from elsewhere and whether this prioritisation tool or process had been evaluated. During recruitment, the research team agreed that a pharmaceutical prioritisation ‘tool’ may be perceived as being electronic and risk not including hospitals that use paper-based tools. The research team, therefore, decided to include a pharmaceutical prioritisation ‘tool or process’ in the online invitation. Eligible NHS health boards and trusts were those with general, acute inpatient services, including paediatric and women’s inpatient hospitals. Specialist organisations such as community mental services, tertiary care-only, cancer, elective-only and inpatient mental health hospitals were excluded, where prioritisation approaches may differ from the general acute setting. The online survey included an information sheet, which participants could read prior to starting the survey. To increase the response rate, the survey was also shared via relevant social media platforms. As decided beforehand, a survey response below 40% required researchers to contact eligible organisations by telephone to obtain answers to the questions in the online survey.

10.1136/ejhpharm-2020-002365.supp1 Supplementary data

### Semistructured interviews

Organisations identified as having a prioritisation tool or process were subsequently invited to participate in a semistructured telephone interview at a time convenient to them. The interview schedule was developed by the research team and reviewed by a practising hospital pharmacist. Interviews were conducted by ASA, PL and MS and included questions about the tool or process used to prioritise patients, how it was used in practice, how it was developed and the perceived advantages and disadvantages of the tool or process. Information about the hospital such as the number of beds and number of employed pharmacy staff was also collected. Interviewees who described a prioritisation tool were asked to email screenshots of the tool and guidelines for its use, to enable understanding of the approach used by the hospital. Consent was obtained from interviewees prior to audio recording the interview. Interviews were transcribed by professional transcribers approved by The University of Manchester. Interviewees were asked if they would like to waive anonymity on behalf of their organisation to allow the name of the hospital or trust to be published. This information can be found in online supplemental table 1.

### Data analysis

A spreadsheet was generated to calculate survey response rates from organisations in all parts of the UK. The interview transcripts were qualitatively analysed using Braun and Clarke’s thematic approach,^14^ to examine themes and patterns within the data and to formulate a description of the methods used to prioritise patients for the delivery of pharmaceutical services across hospital organisations. Tools were broadly categorised by the authors as electronic (E), paper based (P) and paper electronic (PE). Preidentified topics for coding were generated using the interview schedule to categorise data into themes and subthemes that were generated iteratively. New themes emerged as the data were discussed and analysed until data saturation was reached. Highlighted quotes were then tabulated under the theme headings to produce a concise account of how the themes describe the data. Ellipses […] in the quotes indicate pauses or words that have been omitted for clarity. A draft of this manuscript was sent to all interviewees; a small number responded with minor clarifications and these were accepted.

## Results

Eligible representatives such as principal, lead, advanced clinical and chief pharmacists from 130 trusts and health boards responded to the survey, resulting in a response rate of 76.5% (130/170). The survey results revealed that 70 (54%) of these organisations used a tool or process to prioritise clinical pharmacy services.

A total of 36 pharmacists agreed to take part in semistructured interviews, which were conducted between July 2017 and January 2018. Two interviews were excluded from the analysis as whole wards were prioritised in those hospitals, by their typical patients and turnover, instead of individual patients. Twenty-six trusts and health boards were located in England, five in Scotland, two in Northern Ireland and one in Wales. Prioritisation tools ranged in complexity from basic tools that only included high-risk indicators and no stratification, to complex tools that used a number of high-risk indicators and a stratification system to assign a priority level to each patient. The majority of hospitals included in this study (n=23) used tools that included a stratification system to assign a patient priority level.

The introduction of a tool ranged from those that were recently developed (1 month prior to interview) to those that had been in place for up to 10 years prior to interview (median=3 years). A summary table of the prioritisation tools from this study and associated references can be found in online supplemental table 1. Prioritisation tools were mainly developed in-house, assisted by medication safety, medicines information and lead pharmacists within hospitals, often in collaboration with information technology or system teams. In many cases, this was part of broader initiatives to implement electronic patient records in hospitals.

The impetus for development stemmed from patient safety concerns and the need to improve patient flow. Interviewees expressed particular concerns about clinical areas not receiving a routine or comprehensive clinical pharmacy service (such as maternity wards and day surgery units), which on occasion had led to patient safety incidents:

The background to it [tool development] was quite a long time ago now there was a clinical incident …where a patient had…well, basically passed away. (Interview 4, E1)

Many interviewees described difficulties in finding time to review every patient every day due to a shortage of pharmacy staff. Interviewees also described the pressures of meeting the national medicines reconciliation targets and preparing discharge prescriptions before midday for patients to be discharged:

We can’t see every patient every day…so we use that list…to prioritise discharges…so it [the prioritisation tool] allows us to try and identify and improve medicines reconciliation rates as well through using this. (Interview 24, E2)

Based on this reality, pharmacists stated the importance of at least seeing the most acutely unwell patients first:

What’s become really apparent is that we are doing some patients a really significant disservice, because they’re not getting enough time from us. So, this, to me, is less about stopping seeing people who don’t need us, although I think that is important, it’s more about making sure that we’re seeing the people who really do need us. (Interview 36, E2)

In general, determining the priority of a patient relied on identification of certain indicators; these included prescription of high-risk medication that required therapeutic drug monitoring, patients with certain medical conditions, polypharmacy and elderly patients who may be considered complex (box 1). Pharmacists and, in some cases, pharmacy technicians were expected to review patients’ medical condition, age and medication using such indicators to assign a priority level. These were generally stratified as red, amber or green, using the ‘traffic light system’ allowing pharmacists to identify patients in need of immediate or urgent clinical pharmacy input.

Box 1 **Examples of commonly reported indicators included in pharmaceutical prioritisation tools**

- High-risk medicines with suspected toxicity or subtherapeutic effect.
- Medicines requiring therapeutic drug monitoring.
- Medicines reported as omitted or delayed that cause harm (eg, time-critical medicines).
- Medications that cause an increased risk of falls.
- Medicines that cause a QT prolongation or torsade de pointes.
- Intravenous medication (with no specified switch from intravenous to oral/no indicated or defined course/no plan for reduction or discontinuation).
- Strong opiates or opioid dependence.
- Acutely decompensated organ(s).
- Acute kidney injury or newly diagnosed renal impairment.
- Deranged liver function tests elevated from normal (approximately three times the upper limit of normal).
- Age over 75 years old.
- Polypharmacy (eg, on more than 10 regular medicines).
- Extremes of weight (frail/obese).
- Compliance/adherence issues.

Interviewees described a range of prioritisation tools and these were broadly categorised and defined by the research team according to their type. Table 1 provides these categories, our definition and an example from the data. Some were sophisticated tools that assigned priority levels to a patient automatically using algorithms (E1, n=1), highlighted risk-associated indicators (E2, n=10) or relied on reports from other departments to prioritise patients (R, n=1).

**Table 1:** Types of prioritisation tools used in hospitals with examples

Type of tool	Definition	Example
Electronic-based (E) tools, n=12		
E1	Fully integrated electronic tools that use algorithms to assign a priority level to a patient. It can extract patient information from an electronic medical record and may calculate a risk score or assign a priority colour automatically.	*‘…every few minutes a report runs in the background and pulls data for all our patients and calculates them a risk score. And that risk score is based on quite a complicated algorithm which has been developed over the years based on clinical pharmacist input.’* (Interview 4)
E2	Software that allows the user to select any electronically recorded patient indicators which should be flagged for the pharmacist. Indicators may be flagged or highlighted to display whether tasks have been completed or require the attention of the pharmacist (ie, red, amber or green). The software presents itself as a tracking board, electronic whiteboard, dashboard or smart board. Pharmacists will use their prioritisation guidelines to assign a priority level for each patient. This is recorded onto the electronic interface to be accessed anywhere throughout the hospital.	*‘The main tool that we use is something that we call the Pharmacist’s Friend …things that are highlighted on that dashboard…medicine reconciliation state…missed doses of critical medicine, or if they are on high risk medicine…acute kidney injury.’* (Interview 17)
R	Systems in which a report runs in the background to identify patients with preselected risk factor indicators. This usually relies on the pharmacist proactively obtaining results and prioritising patients accordingly.	*‘…because we have an electronic prescribing system, the medicines information department get a report for therapeutic drug levels…if the results are outside the normal range, they would telephone the pharmacist who would then review the patient with toxic levels.’* (Interview 1)
Paper-based (P) tools, n=8		
P	Relies on pharmacist reviewing indicators associated with patients to assign a risk score or priority level and flag potential issues. Usually documented on a handover document or in the patient notes.	*‘…when patients are seen in the big acute medical unit, we document the colour on the front of the drug chart…but then after that we document it on our clinical handover sheets.’* (Interview 6)
Paper-electronic (PE) tools, n=14		
PE	Pharmacists will review patient indicators using paper-based prioritisation guidelines and assign a priority level. The outcome (ie, risk score, priority level) is then recorded on an electronic whiteboard or similar interface. The pharmacy team can access the electronic interface remotely and update the priority of the patient when needed.	*‘So, the actual categorisation of how you would triage is paper based, but we use an app to record it. We have an electronic system, a patient management system.’* (Interview 20)

The majority of pharmacists assigned patients a priority level or score after medicines reconciliation, when all relevant information about a patient’s medication could be taken into account. A few trusts implemented a strategy whereby a patient was assigned a priority level on first contact with pharmacy, and prior to medicines reconciliation, updated later as part of the medicines reconciliation process. Similarly, one trust (Interview 35) took a proactive approach to identify pharmaceutical-related issues prior to medicines reconciliation, in order to intervene during medical postadmission ward rounds. This meant pharmacists spent up to an hour screening patients in the morning. Another trust reported looking into the development of a more advanced technician role to help with the patient prioritisation.

Tools often included a combination of pharmacy service prioritisation and individual patient prioritisation level or risk score. Examples of the former included identification of newly admitted patients or those requiring medicines reconciliation or discharge prescriptions, improving pharmacy service workflow to ensure local targets were met. Furthermore, prioritisation tools were not used in isolation; pharmacists also used handovers and information from ward staff to clarify the types of patients needing greatest pharmacy input.

They [pharmacists] will have a little bit of intelligence from speaking to the ward staff about those patients who are either most unwell or who have obvious issues with their medicines and would go to see those ones first. (Interview 12, PE)

Assigning patients to a prioritisation level depended on a number of indicators, as seen in box 1. A small number of organisations assigned highest priority, complex patients to the most appropriately experienced pharmacist (see Table 2 from online supplemental material for pharmacist band levels based on UK national profiles for pharmacists working in NHS settings):

…red patients should be seen by an 8 [band 8, advanced pharmacist] or the most senior experienced available. The ambers would be largely seen by 7’s and sometimes 6’s. (Interview 12, PE)

Moreover, despite tools providing a standard against which pharmacists should assign a particular priority, pharmacists’ experience and clinical judgement led to variations in the application of the tool:

…pharmacists, generally, are very black and white…that greyness which is ingrained in medicine isn’t in pharmacy, and that greyness that allows you to do it confidently only comes with experience. So there is something around Band 6’s [newly qualified pharmacists], you know, 90% of their patients are red. Whereas I would say if you look at any experienced, trained pharmacist, they’d be able to reduce that. (Interview 31, P)

Interviewees acknowledged the influence of clinical judgement when assigning patients a priority level and highlighted the potential patient safety issues that may arise when less experienced pharmacists use prioritisation tools:

The other problem we’ve noticed…you have got inexperienced pharmacists who maybe don’t actually spot there is an issue and will grade people too low because they’re not aware there’s an issue there. (Interview 8, PE)

Moreover, some pharmacists felt that assigning patients with a higher priority level to the most appropriately skilled pharmacist might lead to deskilling less experienced pharmacists:

…band 8 would cover intensive care anyways; but across the rest of the medical wards…all bands of pharmacists [sic] give a patient the acuity colour…only because it would feel that [it] would de-skill the band 6’s if they were only reviewing green patients. (Interview 6, P)

Therefore, in the majority of organisations, tools were seen as a learning opportunity for less experienced pharmacists and technicians:

Some of the more junior staff were also learning from the band 8’s…because we had the referral sort of system…the band 8’s were then explaining why they were doing certain things, and what the treatment plan needed to be, and what monitoring needed to happen to that patient. And then, that was an educational tool, for, well not just the band 7’s, but also for the technicians. (Interview 33, P)

Interviewees broadly described the benefits of their prioritisation tool, including the ability to target complex patients who needed urgent clinical pharmacist input. From a clinical governance perspective, chief pharmacists and pharmacy managers described how prioritisation tools allowed them to visualise their workforce and clinical demand, providing ‘*a framework and an expectation*’ (Interview 29, P). Prioritisation tools allowed chief pharmacists and pharmacy managers to have oversight to plan and manage workload based on the availability of staff. In some cases, this also allowed managers to delegate tasks based on individual pharmacists’ knowledge and skills:

…as a clinical pharmacy manager, to be able to look through and see which areas may be struggling a bit, and where there may not have been escalations, for whatever reason, be able to approach the team and find out what’s happened or what extra help they need as well. (Interview 10, E2)

So, as well as individual pharmacists managing their own workload, it lets the team leaders manage the work of the whole team and particularly it allows them to target tasks to individuals based on individuals' knowledge and skills. (Interview 9, PE)

In addition, workload prioritisation based on staff availability was thought to improve workflow and create clearer handovers that can contribute to an efficient 7-day service in hospital:

…the advantage of having it on the webpage is it’s live, it’s close enough to be real time…The big advantage it gives us at weekends…is you can edit the view to be as many wards or as few wards as you want…but there’s only two teams working on a weekend so they could see the whole hospital so the team leaders could advocate, we don't normally go to orthopaedics, but there’s a new patient on the orthopaedic ward that’s on high risk drugs, somebody go and sort that out. (Interview 9, PE)

Interviewees described how using a tool provided pharmacists with more confidence in their work prioritisation allowing them to provide pharmaceutical care to complex patients at the right time. This was to reduce the risk of patient harm and improve efficiency in practice. Moreover, in cases where an electronic tool was used, pharmacists were able to retrieve real-time data allowing them to continuously review the patients’ clinical condition and pharmaceutical needs:

The advantages of an electronic system to develop scoring or to assign scoring is, firstly, that it can be done in real time and it reflects what happens continuously…in fact, if someone makes changes to prescribing for a patient after you have turned your back on the patient record, this is immediately reflected in the risk scoring. (Interview 4, E1)

Interviewees also touched on the drawbacks of using a prioritisation tool. These included design issues, the lack in tool sensitivity across certain specialties and time spent using the tool if not all information was accessible. Despite the advantage of speed and real-time access to information when using an electronic tool, some described design issues relating to the ease of access to information:

…it took about 13 or 15 clicks in the system and it took about two to three minutes to actually show that this drug was potentially either being given orally or it was being crushed and putting [sic] down the NG tube. (Interview 1, R)

Despite many advantages of using a prioritisation tool, pharmacists recognised the importance of not over-relying on the tool, which could neglect the importance of one-to-one interactions with patients:

…there are some other soft bits that you’ve lost, a conversation with the patient that make you think, oh, they don’t understand how they’re using their medicines…it can’t know the things that it doesn’t know that the human brain can pick up in conversation. (Interview 9, PE)

Few hospitals had undertaken formal evaluations of their prioritisation tool. Hospitals had mainly collected feedback on the views of pharmacists following use of the tool, in addition to checking the accuracy of data to validate the data fields. Pharmacists briefly described audits or small-scale projects that were either part of pre-registration projects or a master’s thesis. One hospital had carried out detailed evaluation of its prioritisation tool had used data in the electronic patient system as proxy markers for effectiveness of their tool:

…we looked at the change in time between patient admission and creation of the pharmaceutical care plan…I think the other proxy marker was…high-risk medications and how quickly we saw them and how quickly we verified them…because we have electronic prescribing we are able to just interrogate the system for that information. (Interview 4, E1)

## Discussion

There has been a growing interest in the development of pharmaceutical prioritisation tools over recent years.^12 13 15–17^ This is the first study to provide a summary of hospital-based pharmaceutical prioritisation tools and systems across the UK exploring their perceived advantages, drawbacks and limitations. Our study found that just over half of NHS trusts responding to the survey had a prioritisation tool or process to direct their clinical pharmacy services. However, this also means that there are many trusts who operate a traditional model of service delivery. There are some limitations to this study. During the recruitment phase for the interviews, some pharmacists did not clearly distinguish between service and patient prioritisation. This could mean an over-representation in the number of patient prioritisation tools identified in the survey. In addition, conducting interviews with one person from the trust may not necessarily represent the views and experiences of all pharmacists in that trust. Moreover, as not everyone was able to provide us with demographic information, we were unable to explore the potential association of staffing levels and hospital size with the opinions of pharmacists using pharmaceutical prioritisation tools.

This study demonstrates that many UK clinical hospital pharmacy services are finding innovative ways of delivering inpatient services in order to improve workforce efficiency and patient safety. Much of these efforts were borne from a combination of limited staff and increasing workload pressures concerning adherence to national targets.^18 19^ The majority of NHS trusts in this study developed their own prioritisation tool rather than adopted or adapted existing tools. While it is commendable that healthcare professionals are making an effort to find ways to deliver patient services using available resources, adequate staffing and funding is needed to ensure quality of care does not deteriorate. A report addressing this issue highlighted that workforce challenges are ‘the biggest threat facing the health service’, and are already having a direct impact on patient care and staff experience.^20^

A key factor differentiating prioritisation tools used within hospitals is whether they are electronic or paper based. Secondary care providers are expected to deliver digitally enabled care, as envisaged in the NHS Five Year Forward View.^21^ There are current pharmacy management systems, such as PharmAssist^22^ and PharmacyView,^23^ which allow pharmacists to assign prioritisation levels to patients based on the pharmacist’s clinical judgement. This study demonstrated that some hospitals have either set guidelines on how pharmacists should assign patients a priority level, documenting this on their pharmacy management system, or they have developed electronic prioritisation tools using sophisticated algorithms to automatically assign a priority level to patients. Pharmacists in this study emphasised the benefit of electronic prioritisation tools that provide quick access to real-time data.

In a study exploring staff perceptions on workload prioritisation in hospital pharmacy, pharmacists identified the most common barriers to effective clinical prioritisation as lack of time and readily available information.^16^ Another study demonstrated that using an electronic system to identify and prioritise patients was much faster than manual prioritisation and could optimise clinical pharmacist resources.^24^ This suggests that an effective electronic prioritisation tool may better address work demands on hospital pharmacists when assessing the patient complexity. A notable consequence of the active implementation of electronic patient records in hospitals is the reduced number of interactions between the pharmacist and the patient.^25^ This may be especially deleterious during the medicines reconciliation and patient discharge stages of the patient management pathway. Verbal and physical cues add to the pharmacist’s clinical judgement and are likely to influence the impact of patient care.^15 25 26^ This is particularly important when identifying allergies, adherence issues and building a pharmacist–patient relationship.

Similar to previous studies,^16 27 28^ pharmacists recognised the influence of their clinical judgement affecting their confidence when assigning priority levels. Despite this, few hospitals assigned patients to an appropriately skilled pharmacist based on complexity, in line with the cost-effective use of staff practising at their highest skill level.^9^ Using a tool to assess patient priority was viewed as a learning opportunity for pharmacy technicians and less experienced pharmacists. Such clinical prioritisation and decision-making is a skill requiring training and continuous development.^15 16^

Prioritisation of pharmacy services (eg, medicines reconciliation) was often combined with the prioritisation of patients based on their clinical complexity. This makes it hard to differentiate whether the advantages of prioritisation tools were from prioritising pharmaceutically complex patients or from managing staff and patient flow. However, it is possible that it is this combination that provides benefit to clinical pharmacy service managers. A survey of clinical pharmacy staff^16^ found that a wide range of resources were used to prioritise patients and that approaches differed depending on the activity being undertaken. For example, date and time of admission were used to prioritise patients for medicines reconciliation, whereas referrals from the nursing or medical team and ward rounds were used to prioritise patients for clinical review. Prioritisation tools were less commonly used by survey respondents, although this may have changed since that survey was undertaken. That survey, like this study and others,^16 28^ found that assessing patient priority was dependent on the pharmacist’s clinical judgement, the ward and the pharmacy team circumstances. Currently, only two prioritisation tools have been evaluated and internally validated.^29 30^ Further research is needed in order to develop an evidence-based pharmaceutical prioritisation tool that is externally validated.^15^ Similar to the Safer Nursing Care Tool,^4^ it is important to reach a consensus on which criteria should be included in a pharmaceutical care prioritisation tool in order to develop an evidence-based pharmaceutical prioritisation tool rigorously and systematically. An assessment of its usability and acceptability would be imperative to ensure it can be incorporated into the daily workflow routine of pharmacists. Moreover, further research to evaluate such a tool would be needed to ensure it improves patient health outcomes and efficiency in pharmaceutical-led services.

Overall, this study found that just over half of pharmacies within NHS hospitals had developed tools with the aim of prioritising patients based on their complexity. However, the implementation of prioritisation tools depends heavily on context, both within hospitals across the different areas of clinical practice but also across different hospitals due to the influence of wider organisational factors including resources and staffing. Given this variety, it is unclear what is the most appropriate and useful way of prioritising patient pharmaceutical care in order to ensure the improvement of patient health outcomes and further research is warranted.

**What this paper adds**

**What is already known on this subject**

- Hospital pharmacy teams are developing pharmacy prioritisation tools in response to the ever-increasing pressure on healthcare services.
- The aim of pharmacy prioritisation tools is for pharmacy teams to direct their limited resources more effectively and improve patient care by identifying patients who need it most and deploy the right staff, with the right skills at the right time.
- There is limited published literature on the use and development of pharmacy prioritisation tools despite anecdotal knowledge that hospital pharmacies use them.

**What this study adds**

- An understanding of the types, uses, advantages and disadvantages of pharmacy prioritisation tools currently in use.
- The nature of pharmacy prioritisation tools varies across hospitals.
- Few hospitals have formally evaluated their tools and further research is needed to ensure they improve patient health outcomes and efficiency.
- Exploring pharmacy prioritisation tools already in use can help determine what is required to develop an evidence-based tool and national standard or process to determine the prioritisation of patients for clinical pharmacy input.

## Data Availability

All data relevant to the study are included in the article or uploaded as supplementary information. Any queries can be directed to penny.lewis@manchester.ac.uk.
