# Supplementary material for: Patient prioritisation for hospital pharmacy services: current approaches in the UK
Source: Eur J Hosp Pharm. 2020 Dec 1;28(e1):e102–8. doi: 10.1136/ejhpharm-2020-002365 (PMC8640403; doi:10.1136/ejhpharm-2020-002365)
Supplement: Supplementary data [file ejhpharm-2020-002365supp001.pdf]

Supplementary Material

Table 1 Summary table of prioritisation tools used by hospitals in this study

Name	Type of Tool	Service Prioritisation in Tool	Tool Introduction	Tool Development	Rating System	Referring to Pharmacist Seniority Based on Patient Complexity
**Interview 1**	R	Yes	2013	In-house	None	No
**Aintree**	E2	Yes	2010	In-house	Traffic light	Yes (semi-piloted)
**Aneurin Bevan (Royal Gwent Hospital)**	E2	No	Under development	In-house	Flags for 2 conditions/medication	No
**Ayrshire and Arran Health Board**	E1	Yes	2013	In-house	Traffic light	No
**Interview 5**	P	No	2017	Adapted from Hickson et al., 2017 (1)	Level 1-3	Yes
**Brighton and Sussex**	P	Yes	2017	Adapted from Newcastle's model	Traffic light	No
**Chesterfield**	PE	Yes	2017	In-house	Level 1-3	No
**County Durham and Darlington**	PE	Yes	2012	In-house	Traffic light	No
**Doncaster and Bassetlaw**	PE	Yes	-	In-house	Level 1-3	Yes – competency-based
**Dorset County**	E2	Yes	2015	In-house	Traffic light for medicines reconciliation; pink for high-risk	No

					medicines; red for unverified orders	
**Fife**	PE	Yes – medicines reconciliation only	2014	Adapted from Greater Glasgow & Clyde (2)	Traffic light	Yes
**Greater Glasgow and Clyde**	PE	Yes	-	In-house, originally based on Tayside's processes	Traffic light	No
**Leeds Teaching Hospital Trust**	PE	Yes	2014	In-house	Level 1 & 2	No
**Luton and Dunstable**	E2	Yes	2014	Adapted from Royal Cornwall's model	None	No
**Northumbria Healthcare**	PE	Yes – medicines reconciliation only	2017	Adapted from County Durham and Darlington NHS Trust's model	Level 1-4	Yes
**Royal Cornwall**	E2	Yes	2016	In-house (two tools):<br><br>Weekday tool<br><br>Weekend acuity tool adapted from Ayrshire and Arran's model	Traffic light<br><br>Acuity score 0-10	No
**Royal Devon and Exeter**	E2	Yes	2014	In-house	None	No
**Interview 19**	P	Yes – mainly service prioritisation process; only uses acute	-	In-house	None	No

		kidney injury and age as risk factors for prioritisation.				
**South Eastern Health and Social Care Trust**	PE	No	2017	In-house but initially adapted and developed from Greater Glasgow & Clyde (2)	Traffic light	No
**Southend University Hospital**	E2	Yes – service prioritisation first, followed by clinical complexity of patients	2016	In-house	Flags with letters	No
**Interview 22**	P	No	2007	In-house	None	No
**Interview 24**	E2	Yes	2014	In-house	None	No
**Interview 25**	PE	Yes	2013	Adopted from other health board's	Traffic light	No
**Interview 26**	P	-	2017	Adapted from Greater Glasgow & Clyde (2)	Red, blue, green	No
**Interview 27**	PE	Yes	2013	In-house	None	No
**Interview 28**	E2	-	2013	Adapted from Greater Glasgow & Clyde (2)	Traffic light	No
**Interview 29**	P	Yes	2014	In-house	None	No
**Walsall**	PE	-	2016	Adapted from South West model	Level 1-3	No
**Interview 31**	P	Yes	2016	In-house	Traffic light	No

**York NHS Trust**	PE	Yes	2014	In-house	None	-
**Central Manchester Foundation Trust**	P	Yes	-	In-house	P1-P4	Yes (Surgical Unit)
**South Manchester**	PE	Yes	2013	In-house	Level 1-3	Yes
**Interview 35**	PE	-	2016	In-house	None	No
**Newcastle upon Tyne NHS Foundation Trust**	E2	-	2012	In-house	None	No

(E) Electronic; (P) Paper-based; (PE) Paper-Electronic. E1 = Fully integrated electronic tools that use algorithms to assign a priority level to a patient; E2 = Software that allows the user to select any electronically recorded patient indicators which should be flagged for the pharmacist. The software presents itself as a tracking board, electronic whiteboard, dashboard or smart board. Pharmacists will use their prioritisation guidelines to assign a priority level to each patient; R= Systems in which a report runs in the background to identify patients with pre-selected risk factor indicators; P= Paper-based tool that relies on pharmacist reviewing indicators associated with patients to assign a risk score or priority level. This is usually documented on a handover document or in the patient notes; PE= A paper-electronic tool where pharmacists will review patient indicators using their prioritisation guidelines and assign a priority level. The outcome is then recorded on an electronic whiteboard or similar interface.

* Interviewees who chose to waive anonymity on behalf of their organisation have been named in the table, those that did not have been assigned a number. Any information the author was unable to obtain from the participant has been presented in the table as a dash.

Table 2 – Pharmacist band levels based on the UK national profiles for pharmacists working in NHS settings (3)

**Pharmacist Profile Label**	**Band Level**
Pharmacist Entry Level Pre-registration Pharmacist	5
Pharmacist	6
Pharmacist Specialist	7
Pharmacist Advanced	8a-b
Pharmacist Team Manager	8b-c
Pharmacy Consultant	8b-d
Professional Manager Pharmaceutical Services	8c-9

Definitions

NHS Trusts/Health Boards: An NHS Trust is an organisation that is a legal entity set up by order of the Secretary of State to the National Health Service Act 2006 to provide goods and services for the purposes of the health service in England. (4) The equivalent of an NHS Trust that is based in Northern Ireland, Scotland and Wales is referred to as a Health Board or a Health and Social Care Board. (5)

References

1. Hickson RP, Steinke DT, Skitterall C, Williams SD. Evaluation of a pharmaceutical assessment screening tool to measure patient acuity and prioritise pharmaceutical care in a UK hospital. European Journal of Hospital Pharmacy. 2017;24(2):74-9.
2. NHS Greater Glasgow and Clyde. Pharmacy Prioritisation and Referral. PostScriptAcute NHS Greater Glasgow and Clyde: NHS Greater Glasgow and Clyde,; 2014 [Available from: http://www.ggcprescribing.org.uk/media/uploads/postscript_acute/ps_acute_issue_17_june_2014.pdf.
3. NHS Employers. National Profiles for Pharmacy [Available from: https://www.nhsemployers.org/-/media/Employers/Documents/Pay-and-reward/Pharmacy-final-version-Dec-18.pdf.
4. NHS. NHS Trust NHS Business Definitions [Available from: https://www.datadictionary.nhs.uk/data_dictionary/nhs_business_definitions/n/nhs_trust_de.asp?shownav=1.
5. NHS Health Education England. UK Health Systems [Available from: https://www.healthcareers.nhs.uk/working-health/uk-health-systems.
